# Buffering and Amplifying Transcriptional Noise During Cell Fate Specification

**DOI:** 10.3389/fgene.2018.00591

**Published:** 2018-11-29

**Authors:** Elizabeth A. Urban, Robert J. Johnston

**Affiliations:** Department of Biology, Johns Hopkins University, Baltimore, MD, United States

**Keywords:** transcriptional bursting, expression noise, cell fate, Waddington landscape, MS2 hairpin, smFISH

## Abstract

The molecular processes that drive gene transcription are inherently noisy. This noise often manifests in the form of transcriptional bursts, producing fluctuations in gene activity over time. During cell fate specification, this noise is often buffered to ensure reproducible developmental outcomes. However, sometimes noise is utilized as a “bet-hedging” mechanism to diversify functional roles across a population of cells. Studies of bacteria, yeast, and cultured cells have provided insights into the nature and roles of noise in transcription, yet we are only beginning to understand the mechanisms by which noise influences the development of multicellular organisms. Here we discuss the sources of transcriptional noise and the mechanisms that either buffer noise to drive reproducible fate choices or amplify noise to randomly specify fates.

## Introduction

Cell fate specification during development is often thought of as a highly reproducible process where tight regulation of gene expression determines precise cell fates. These robust, reproducible fates are driven by cell lineage history and signaling. A beautiful example of lineage-driven cell fate specification occurs in the nematode *Caenorhabditis elegans.* Nearly all of the cells of the worm derive from stereotypical division patterns and gene expression that have been very well-mapped (Sulston et al., 1983; Maduro, 2010). For example, the ASEL/R neurons are derived from a distinct lineages that are regulated by a network of transcription factors and microRNAs (Hobert et al., 2002; Johnston and Hobert, 2003, 2005; Chang et al., 2004; Johnston et al., 2005, 2006; Poole and Hobert, 2006; Sarin et al., 2007; Cochella et al., 2014). Conversely, one of the best understood paradigms for signaling-driven development is observed in the eye of the fruit fly *Drosophila melanogaster.* In the fly eye, precise and progressive signaling cues determine retinal cell fates, generating a near-crystalline pattern of ommatidia (Wolff and Ready, 1991; Kumar, 2011, 2012). All photoreceptors develop from the same pool of undifferentiated progenitor cells (Kumar, 2012). The final photoreceptor to develop, the R7, is generated through combinatorial Notch, RAS, and EGFR signaling from the other photoreceptor subtypes (Kumar, 2012). The transformation of a pool of undifferentiated progenitor cells into 800 ommatidia arranged in a crystalline pattern across the retina highlights the importance of signaling as a mechanism to determine robust cell fates.

Lineage and signaling cues provide a framework for the energy landscape of cell fate specification first described by Waddington (1957). In Waddington’s energy landscape, “hills” and “valleys” represent developmental energy potential. These geographical landmarks are used to guide cells toward terminal differentiation. Lineage and signaling inputs push cells into valleys of low potential energy, thereby restricting them to specific fates (Waddington, 1957).

**FIGURE 1:**
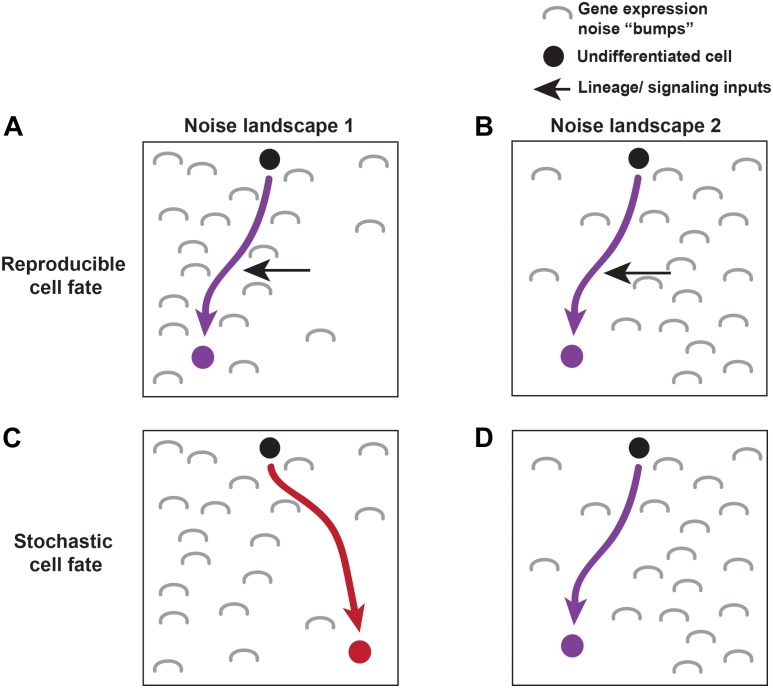
Lineage, signaling, and noise make up the molecular environment driving cell fate specification. An undifferentiated cell (black) moves towards its terminal cell fate based on its molecular landscape (described by Waddington’s energy landscape). Gene expression noise effects the landscape through which cells differentiate. Two different noise landscapes are shown (**A,C** vs. **B,D**). Noise is depicted by gray “bumps.” Reproducible fates are able to overcome noise in both landscapes by utilizing lineage and signaling cues to push them towards a particular fate **(A,B)**. Other cells choose their fate stochastically, where noisy inputs shape the molecular environment driving the stochastic fate decision **(C,D)**.

The road to differentiation isn’t always smooth. Lineage and signaling must overcome molecular noise to drive cell fates. **Gene expression noise** is characterized by differences in the level of gene expression between cells of the same type. It arises due to random fluctuations in the level of mRNA or protein expressed at a given time in an individual cell. Noise roughens the road in Waddington’s developmental landscape, generating “bumps” in gene expression that lineage and signaling cues often override (Balazsi et al., 2011) (Figure 1). However, sometimes these bumps are employed during development to generate a “fork” in the road, causing a cell to randomly fall into one of two fates. Slight variations in the level of noise change the contours of the fork, steering the cell toward one of the fates at a particular frequency. This random choice between fates is called **stochastic cell fate specification** (Figure 1). Together, stochastic fate specification complements lineage- and signaling-based mechanisms to further diversify cell types during development (Johnston and Desplan, 2010).

In single-celled organisms, stochastic cell fate choices generate cellular diversity and facilitate survival in adverse conditions. In the bacterium *Bacillus subtilis*, about 10% of cells transiently enter a competent state in which they are able to take up DNA, while the other 90% remain in a dormant state. This diversity allows the competent population to survive under stressful conditions (Maamar and Dubnau, 2005; Maamar et al., 2007). These two cell populations are genetically identical and are exposed to the same external cues, indicating that this stochastic fate choice is independent of initial genetic and environmental conditions. Rather, intrinsic gene expression noise causes isogenic populations of cells to exhibit varying levels of expression of numerous genes, tipping the balance toward one fate or the other within individual cells (Losick and Desplan, 2008; Raj and van Oudenaarden, 2008; Enver et al., 2009; Balazsi et al., 2011).

**FIGURE 2:**
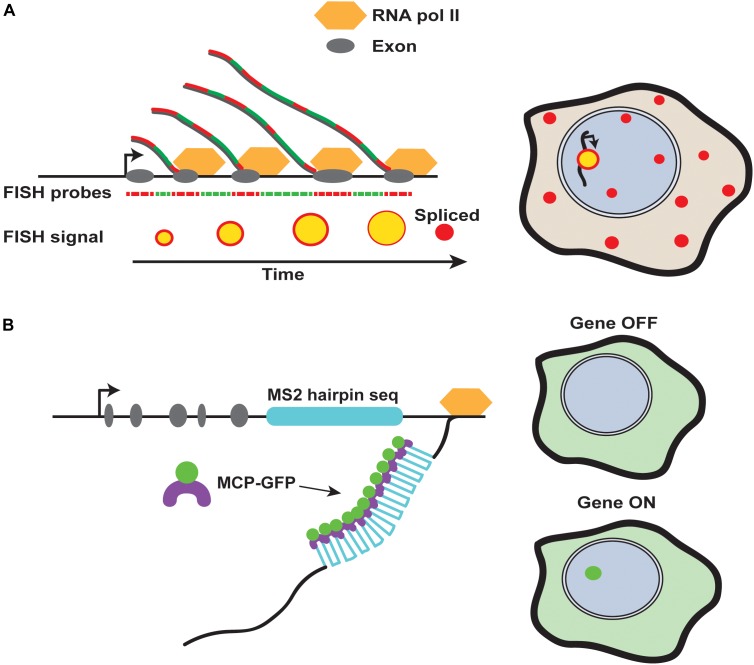
Methods for detection of nascent RNA transcripts. **(A)** Fluorescence *in situ* hybridization tracks transcriptional dynamics. Different colored fluorescent probes against intronic and exonic regions of a gene can be alternated to monitor the progression of transcription in fixed cells. Sites of nascent transcription will contain both fluorophores, while mature transcripts will be labeled by only one fluorophore due to splicing. **(B)** The MS2 system allows for detection of transcripts in living or fixed tissues. The nascent site of transcription is detected by binding of MS2 coat protein (MCP) to MS2 RNA hairpins, which can be incorporated into transgenes and endogenous loci in multiple copies.

Although noise plays a role in stochastic cell fate specification, the mechanisms driving these decisions are poorly understood. In addition to studies performed in bacteria and cultured cells, recent advances in imaging allow the study of stochastic fate choices in multicellular organisms (Box 1 and Figure 2). In this review, we discuss gene expression noise and transcriptional bursting, and examine how these sources of noise are buffered in deterministic fate choices and amplified in stochastic cell fate decisions. Understanding the role of gene expression noise in cell fate decisions is crucial for determining when and how these systems go awry.

**Box 1 |** Studying the dynamics of transcriptional bursting. With recent advances in imaging technologies, we can now study transcriptional bursting in greater detail using techniques such as single molecule RNA fluorescent *in situ* hybridization (smFISH) and the MS2/MCP system (Bertrand et al., 1998; Gregor et al., 2014; Lenstra et al., 2016) (Figure 2). Each of these techniques provides unique insight into the kinetic parameters regulating transcriptional bursting.

smFISH uses fluorescent DNA probes to label nascent RNA transcripts. The amount of RNA produced at the nascent site of transcription is reflected in the fluorescence intensity. Therefore, the elongation rate, length of a transcript, and probe density are used to calculate the exact number of RNA molecules produced (Little et al., 2013; Zoller et al., 2018). Even more information can be extracted from multi-color FISH experiments. For example, the 5′ and 3′ end of a transcript can be labeled in two different colors, or introns and exons can be differentially labeled, allowing the temporal state of transcription to be analyzed in fixed tissues (Little et al., 2013; Zoller et al., 2018) (Figure 2A).

The MS2/MCP system provides a complementary system to study transcriptional bursting parameters. Using this system, multiple copies of a sequence coding for MS2 RNA hairpins are incorporated into a gene of interest (Bertrand et al., 1998) (Figure 2B). Upon transcription, these hairpin sequences are recognized by the MS2 coat protein (MCP). MCP is directly tagged with GFP and expressed at low levels in the cells or tissue of interest. When the hairpins are expressed, MCP-GFP binding to the transcript allows nascent transcription to be monitored in real time in living cells and tissues (Gregor et al., 2014). Thus, dynamics of transcription, such as the burst frequency, duration, amplitude, and total amount of RNA can be determined.

## Sources of Noise in Gene Expression

Gene expression noise arises in many different ways, and the extent of noise varies dramatically among different genes and organisms (Balazsi et al., 2011; Suter et al., 2011). Gene expression noise has two major types: **extrinsic** and **intrinsic** noise (Elowitz et al., 2002). Extrinsic noise arises from environmental perturbations surrounding the cell, and within the cell, such as changes in the local distribution and concentration of general transcription factors or other proteins. Extrinsic noise varies from cell to cell but has the potential to affect all genes, and is therefore a **gene-independent characteristic**. Conversely, intrinsic noise can be attributed to things such as variation in transcription factor and chromatin modifier binding at individual gene loci. Thus, intrinsic noise is considered a **gene-dependent characteristic** because its effects vary from gene-to-gene and from cell-to-cell (Gregor et al., 2014; Fujita et al., 2016).

**FIGURE 3:**
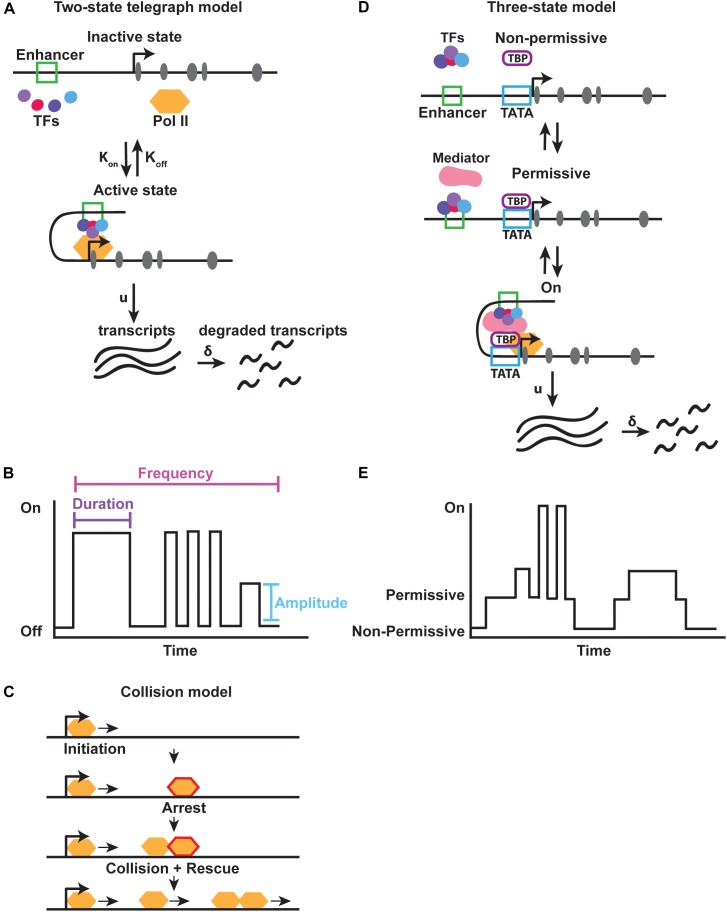
Models for transcriptional bursting. **(A)** The two-state bursting model suggests that a gene flips between an “inactive” and “active” state at a particular rate, K_on_ and K_off_. In the “active” state RNA transcripts are produced at a rate (u) and degraded at a rate (δ). **(B)** Transcriptional bursting parameters. Each burst occurs for a particular duration (i.e., period of time) with a distinct amplitude (i.e., strength) and at a particular frequency. Adapted from Raj et al. (2006). **(C)** A modified two-state collision model suggests that intrinsic interactions between RNA polymerase and DNA are sufficient to create transcriptional bursts. Polymerase arrest, followed by collision and rescue allow for individual bursts to occur. Adapted from Tantale et al. (2016). **(D,E)** The three-state bursting model suggests that a slow burst cycle and fast burst cycle are controlled by the binding of TATA binding protein (TBP) to the promoter, and subsequent recruitment of the co-activator Mediator, allowing a gene to be in either a non-permissive, permissive, or active state of transcription.

The majority of gene expression noise arises from stochasticity in mRNA production due to the random binding of transcription factors and other transcriptional machinery to the DNA, as well as due to the turnover of mRNA and protein (McAdams and Arkin, 1997; Elowitz et al., 2002; Ozbudak et al., 2002; Swain et al., 2002; Raser and O’Shea, 2004). Binding and dissociation events at the enhancer or promoter occur with particular rates, K_on_ and K_off_ respectively (Figure 3A). These rates are largely dependent on protein availability and the presence of other bound proteins. Extrinsic fluctuations produce variability in the local concentrations of proteins, leading to alterations in the probability of protein binding over time. Gene expression noise also arises from variation in the transcription initiation rate (μ) and mRNA degradation rate (δ) (Figure 3A). For highly regulated genes, noise is strongly buffered. For example, noise is reduced by controlling the degradation rate of mRNA. mRNAs with faster turnover rates have lower noise, because the amount of protein that can be translated before the transcript is degraded is limited (Swain, 2004; Cai et al., 2006; Singh, 2011). While gene expression noise also arises from translation, we focus here on the role of intrinsic transcriptional noise, in particular how transcription occurs in non-continuous bursts, referred to as **transcriptional bursting** (Ross et al., 1994; Peccoud and Ycart, 1995; Newlands et al., 1998; Takasuka et al., 1998; Golding et al., 2005).

### Two-State Models of Transcriptional Bursting

Transcriptional bursting occurs when a gene promoter fluctuates between an “on” and “off” state for different periods of time. Each time the promoter switches to an “on” state, a burst of transcription is produced (Peccoud and Ycart, 1995; Gregor et al., 2014; Kumar et al., 2015). The frequency, duration, and amplitude of bursts determine the total amount of RNA produced from a particular gene (Figure 3B). In this manner, transcriptional bursting is a mechanism that produces gene expression noise. This noise is often described by the amount of variation in gene expression seen among cells of a population.

There are two major models of transcriptional bursting: the two-state and three-state models (Figure 3). Multiple versions of the two-state model have been proposed, but the **two-state telegraph** **model** is most commonly used. In the two-state telegraph model first described by Peccoud and Ycart, a promoter’s on/off state is controlled by intrinsic variability in transcription initiation (Peccoud and Ycart, 1995; Lenstra et al., 2016). In this model, the promoter for a gene is in an active or inactive state for different periods of time (Figure 3A). While in the “on” state, the gene produces bursts of transcripts.

*In vitro* single molecule imaging of RNA polymerase and mRNA production has provided evidence for a second two-state model known as the **two-state collision model**. In the collision model, the “off” state is determined by RNA polymerase arrest. Transcription is then rescued back to the “on” state by collision of the next polymerase, indicating that bursting can be produced by general transcriptional machinery dynamics alone (Fujita et al., 2016) (Figure 3C). Although arrest and release can be visualized *in vitro*, whether this pausing and collision actually occurs *in vivo* is unknown. It is possible that promoter proximal pausing could provide a long enough arrest to allow collision and bursts.

Evidence for the two-state collision model stems from *in vitro* work, but a related mechanism for bursting has been observed in bacteria. Bacterial DNA is packaged into topologically constrained loops (Postow et al., 2004; Hardy and Cozzarelli, 2005). As RNA polymerase transcribes these loops, it induces positive supercoiling ahead of the transcription machinery and negative supercoiling behind the transcription machinery. As positive supercoiling accumulates ahead of the polymerase, initiation is blocked, and elongation is slowed. Transcription remains arrested until gyrase (topoisomerase II) relieves the positive supercoiling, producing a burst of transcription until the next arrest. These observations suggest that the binding of gyrase to DNA promotes transcriptional bursts by inducing RNA polymerase II pause release (Chong et al., 2014). By combining the *in vitro* findings about the collision model, with *in vivo* work in bacteria, it is interesting to consider the possibility of a similar mechanism of pause and release creating bursts in eukaryotes as well.

Eukaryotic DNA is packaged into topologically associated domains (TADS) (Jackson and Pombo, 1998; Ma et al., 1998; Dixon et al., 2012; Nora et al., 2012; Sexton et al., 2012). Although the direct mechanisms driving TAD formation are still being investigated, one common model is the loop extrusion model, where DNA is fed through cohesin as a loop, and is halted at boundaries containing CTCF proteins. Simulation work by the group of Racko and colleagues has shown that transcription within a region of DNA, constrained by cohesin on each end, can create the spatial constraint necessary to promote negative supercoiling accumulation within intra-TAD structures (Nasmyth, 2011; Sanborn et al., 2015; Dixon et al., 2016; Fudenberg et al., 2016; Stigler et al., 2016; Racko et al., 2018). Whether this negative intra-TAD supercoiling is seen *in vivo* is unknown, but with similar physical constraints to bacterial chromosomes, it will be interesting to see if this mechanism of bursting is conserved and how it relates to TAD formation.

While the telegraph and collision models describe general mechanisms controlling transcriptional bursting across all genes (i.e., gene-independent), they do not explain how genes in the same nucleus exhibit different kinetics. Promoter architecture, chromatin state, enhancer-promoter interactions, transcription factor copy number, and binding kinetics control different bursting parameters such as amplitude, duration, and frequency of bursting from individual loci (Skupsky et al., 2010; Suter et al., 2011; Dar et al., 2012; Larson et al., 2013; Senecal et al., 2014; Bartman et al., 2016; Fujita et al., 2016) (Figure 3B). These different parameters ultimately determine the levels of RNA for each gene and thus the amount of noise within a population, which are detected by techniques such as single molecule Fluorescent *in situ* hybridization and the MS2 hairpin system (Box 1 and Figure 2). We explore these different contributions to bursting in more detail below.

### Three-State Model of Transcriptional Bursting: Promoter Contributions

As the landing site for the general transcription machinery and RNA polymerase, the core promoter plays a key role in regulating transcriptional bursting. Promoter architecture varies across the genome. Studies in mouse B-cell culture elegantly showed that unique core promoter elements differentially and precisely regulate either the burst size or frequency (Hendy et al., 2017). For example, the TATA box of the MHC class 1 gene PD1 regulates both burst size and frequency, the Initiator element regulates only frequency, and the transcription factor Sp1 binding site regulates only size. These differences in promoter composition allow for tissue-specific bursting effects, which can be modulated by *trans*-acting factor concentrations and binding affinities (Senecal et al., 2014; Hendy et al., 2017).

**FIGURE 4:**
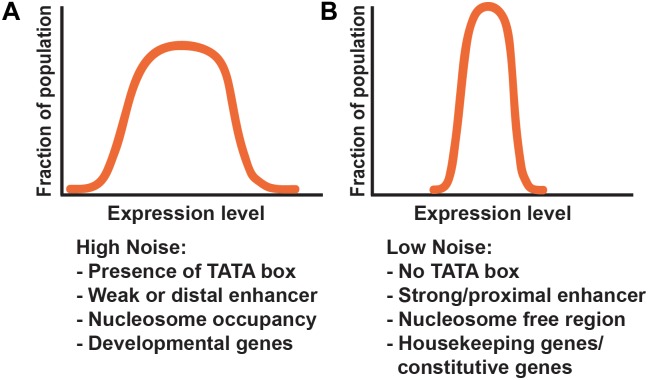
Gene architecture contributes to noise. **(A,B)** Graphs represent theoretical frequency distributions of gene expression levels across a population of cells. Common components associated with differential effects on noise are depicted below each distribution, and include promoter architecture, enhancer strength and position, and chromatin state. **(A)** Genes with high noise generally show a broad distribution of expression levels. Common factors associated with high noise include the presence of a TATA box, weak or distal enhancer elements, and high nucleosome occupancy. **(B)** Low noise-producing genes have a tighter distribution of expression. Common factors associated with low noise include the absence of a TATA box, strong or proximal enhancers, and nucleosome free regions.

The TATA box is a critical determinant of gene expression noise. Genes lacking a TATA box are associated with lower noise, while the presence of a TATA box promotes higher noise (Figure 4). In general, promoters of housekeeping genes lack TATA boxes, suggesting a mechanism by which constitutively and highly expressed genes buffer gene expression noise throughout development (Blake et al., 2006; Hornung et al., 2012; Larson et al., 2013; Zabidi et al., 2015; Tantale et al., 2016; Hendy et al., 2017).

How does the presence of a TATA box promote higher gene expression noise? A study examining the roles of the TATA binding protein (TBP) and Mediator complex suggested a **three-state bursting model** (Tantale et al., 2016) (Figure 3D). Binding of TBP to the promoter induces the promoter to switch from a non-permissive to a permissive state. This generates long periods of inactivity followed by periods of high activity (Figures 3D,E). The binding of the co-factor Mediator to TBP and the promoter then enables rapid on/off cycling of the promoter. Retention of TBP at the promoter may allow rapid re-initiation of transcription by Mediator. This multi-state model has been used to describe bursting in mammalian cells where a non-permissive, or refractory, state is seen between bursts that is potentially driven by chromatin remodeling events (Harper et al., 2011; Suter et al., 2011). In some cells, a gene may be poised for activation, while in other cells, the gene may remain in a non-permissive refractory state (Harper et al., 2011). This refractory period increases noise, creating heterogeneities within a population.

Additional work studying the role of promoters in transcriptional bursting has brought up conflicting data about whether promoter elements buffer or foster noise. In studies by Hendy and colleagues, mutation of core promoter elements increased noise, indicating that promoter elements buffer and tune noise naturally (Hendy et al., 2017). Since many factors bind the promoter, it makes sense that promoters would be regions where noise is buffered by higher binding affinities (i.e., K_on_). In contrast, other experiments suggest that promoters have naturally evolved to produce noise. For example, synthetic *Escherichia coli* promoters were generated in the lab through directed evolution. These artificially evolved promoters drove gene expression at similar levels to their endogenous promoters (Wolf et al., 2015). However, the promoters evolved in the lab produced lower noise than promoters naturally found in *E. coli* (Bury-Mone and Sclavi, 2017). Taken together, these studies suggest that evolutionary pressures may both promote and reduce noise at the level of the promoter sequence, which in turn tunes noise for specific genes (Wolf et al., 2015).

### Enhancer Contributions

In addition to promoter architecture, enhancer-promoter interactions act as gene-dependent regulators of transcriptional bursting. Enhancers are bound by gene-specific combinations of transcription factors to control spatial and temporal expression (Levine, 2010; Buecker and Wysocka, 2012). In addition to binding specific transcription factors, enhancers also assist in recruiting the general transcriptional machinery. What we know about enhancer function has mainly been assessed through reporter assays and transgenes. These approaches give a broad view of expression, but the role of endogenous enhancers in transcriptional bursting at the single cell level is less understood (Bulger and Groudine, 2011; Spitz and Furlong, 2012).

One mechanism by which enhancers affect bursting is through enhancer-promoter looping (Lewis, 1978; Lettice et al., 2003; Fukaya et al., 2016). Kinetic fluctuations in the cellular environment, such as changes in the concentration and distribution of transcription factors, change the probability of enhancer-promoter contact from cell-to-cell. These changes in enhancer-promoter interactions lead to differences in bursting between cells (Lenstra et al., 2016; Tantale et al., 2016).

**FIGURE 5:**
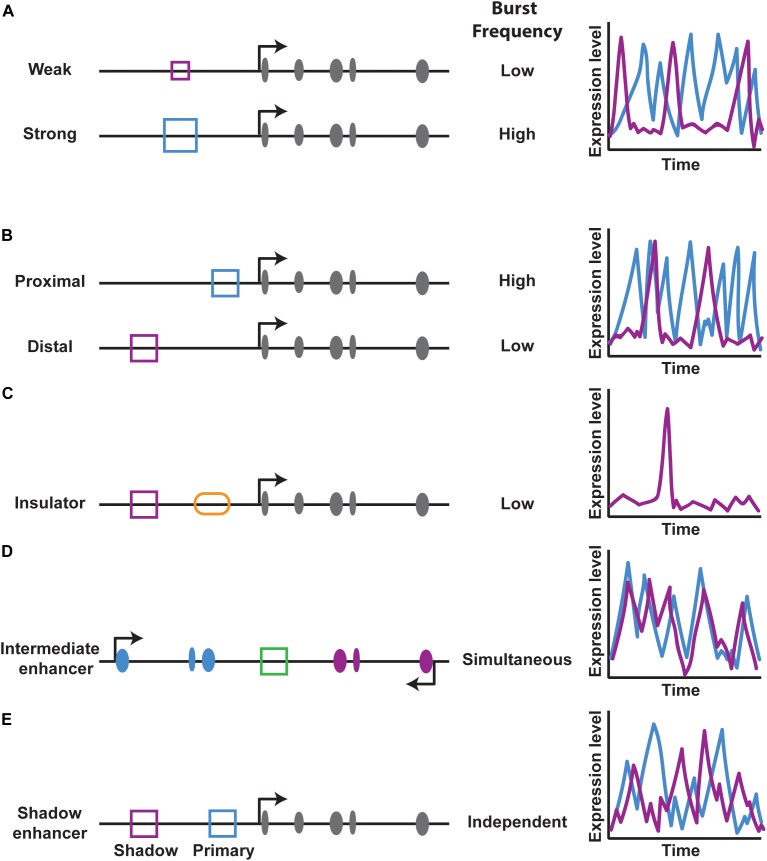
Enhancer effects on transcriptional burst frequency. **(A,B)** Enhancer strength and distance regulate transcriptional burst frequency. Schematics on the left of gene loci depict exons in gray, the enhancer type (blue or purple square) and other *cis*-regulatory elements. Graphs on the right show theoretical burst frequencies from each enhancer over time. **(C)** The presence of an insulator element (orange oval) between an enhancer-promoter pair reduces transcriptional burst frequency. **(D,E)** An enhancer placed between two promoters acts on both promoters simultaneously, while the presence of two enhancers that act on the same promoter act independently, as in the case of shadow enhancers.

The effect of a particular enhancer on bursting is also dependent upon enhancer strength, or how well it can find and loop to its promoter. Weak enhancers drive a lower burst frequency than strong enhancers (Fukaya et al., 2016) (Figure 5A). Additionally, enhancers that are closer to promoters have higher burst frequencies compared to distal enhancers, indicating that proximity increases the probability of an enhancer finding its promoter (Fukaya et al., 2016) (Figure 5B). DNA looping co-factors known as insulators block interactions between particular regions of DNA (Geyer and Corces, 1992; Dorsett, 1993; Bell et al., 2001). An insulator element inserted between an enhancer and promoter reduces bursting by blocking the enhancer-promoter interaction (Fukaya et al., 2016) (Figure 5C). In contrast, tethering an enhancer to a promoter increased the frequency of bursts (Bartman et al., 2016). Thus, enhancer-promoter interactions make critical contributions to transcriptional noise by controlling burst frequency.

While the study of simple enhancer-promoter interactions provides important insight into the effects of enhancer looping on bursting, many genes have a more complex regulatory logic. For example, some enhancers activate multiple genes and some genes are regulated by multiple enhancers, raising two questions: can an enhancer simultaneously activate multiple promoters, and can multiple enhancers simultaneously regulate a single promoter? To address the first question, Fukaya and colleagues examined the activity of an enhancer placed between two promoters (Fukaya et al., 2016) (Figure 5D). The enhancer simultaneously activated both promoters in the *Drosophila* embryo, leading to coordinated bursting (Fukaya et al., 2016) (Figure 5D).

While an enhancer can act on multiple promoters at once, can multiple enhancers act on the same promoter simultaneously? To investigate this second question, Bothma and colleagues looked at how primary and shadow enhancers regulate bursting of a single gene and found that these enhancers do not act simultaneously, indicating that the overall bursting parameters of a gene are a result of an additive effect of combined enhancer inputs (Bothma et al., 2015) (Figure 5E).

Complex networks of enhancers, such as super-enhancers, regulate genes involved in cell type specification and development (Hnisz et al., 2017). Super-enhancers consist of a collection of enhancers with high densities of transcription factors that regulate a gene. Simulation studies of super-enhancer regulation of transcription by Hnisz and colleagues has suggested that the increased strength of super-enhancers should lead to high burst frequencies, creating constitutive gene expression. One potential mechanism by which super-enhancers could increase transcriptional activity is through phase transitions, bringing all enhancers in close proximity to their promoter through networks of cooperative binding. This may explain a mechanism by which gene expression noise is reduced at genes involved in processes regulating cell type, as these are the genes often associated with super-enhancers (Hnisz et al., 2017). Whether all enhancers are acting simultaneously or independently is still unknown, but it will be interesting to see if super-enhancers actually form phase transitions *in vivo*, and how each enhancer contributes directly to transcriptional activity. Together, this model of phase transition of super-enhancers, in addition to the findings of Fukaya et al. (2016) suggest that beyond the role of enhancers, chromosome topology plays an integral role in the regulation of gene expression and enhancer-promoter interactions.

### Chromatin Contributions

The studies described above indicate that enhancer-promoter looping is a critical regulator of transcriptional bursting and thus gene expression noise. Higher-order chromatin studies have revealed that, beyond enhancer-promoter contacts, chromosomes are organized into topologically associated domains, or TADs (Dixon et al., 2016). Within these TADs, DNA contacts are made and facilitated by insulator and architectural proteins such as CTCF and cohesin (Dowen et al., 2014). Depletion of CTCF or cohesin subunits result in intra-TAD contact changes, associated with misregulation of gene expression (Handoko et al., 2011; Seitan et al., 2013; Sofueva et al., 2013; Zuin et al., 2014; Ing-Simmons et al., 2015; Dixon et al., 2016). Studies in mammalian cells have revealed that intra-TAD CTCF binding promotes and stabilizes enhancer-promoter interactions to regulate gene expression. These stabilized interactions result in a decrease in transcriptional bursting and noise (Ren et al., 2017).

The role of the architectural insulator protein CTCF in controlling enhancer-promoter interactions suggests that chromatin state and topology are also important regulators of transcriptional bursting. Hendy et al. (2017) have shown that the level of chromatin compaction and the kinetics required for heterochromatin opening are rate limiting steps in transcriptional bursting. Promoters with nucleosome-free regions downstream of the transcriptional start site display low gene expression noise (Figure 4). In contrast, promoters with higher nucleosome occupancy are associated with high noise, suggesting that chromatin remodeling during transcription initiation may contribute to bursting (Figure 4) (Cairns, 2009). Studies in cell culture showed that increasing acetylation at target transgenes resulted in an increase in burst frequency. These findings indicate that H3K27 acetylation around the promoter may act as a possible regulator of transcriptional burst frequency (Nicolas et al., 2018).

**FIGURE 6:**
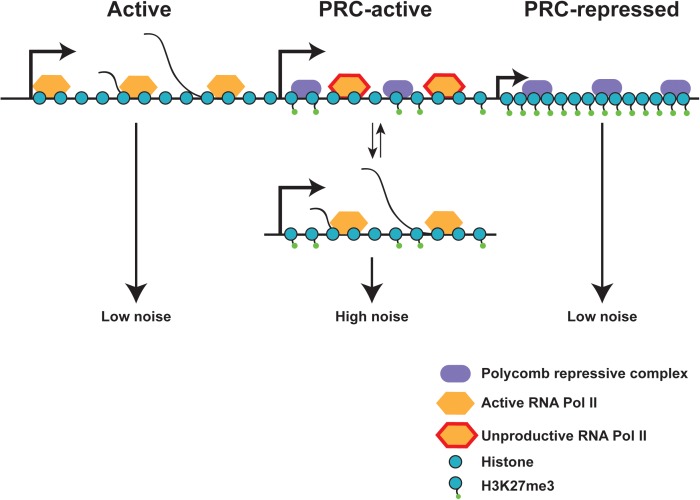
Chromatin state effects on gene expression noise. Fully active and fully polycomb-repressed genes show low gene expression noise, while polycomb-active genes show high expression noise. Polycomb-active genes are marked by both polycomb repressive marks (H3K27Me3) and active RNA polymerase II. These polycomb-active genes are often found in close proximity to polycomb-repressed genes, and may flip between the active and inactive state, increasing gene expression noise. Local heterochromatin spreading due to proximity of polycomb-repressed genes may aid in the switch between active and repressed states. Adapted from Kar et al. (2017).

While H3K27 acetylation at the promoter is an activating histone mark and increases bursting, it is interesting to consider how repressive marks, such as H3K27 tri-methylation affect bursting. H3K27me3 is spread to create repressive heterochromatin by the Polycomb repressive complex (Schuettengruber et al., 2017). Despite their opposing roles, some genes contain both active RNA polymerase (CTD Serine 5, 7, and 2 phosphorylated) and repressive H3K27me3. Genes containing these conflicting markers show higher levels of gene expression noise when compared to fully active genes or fully Polycomb-repressed genes (Kar et al., 2017) (Figure 6). These genes with higher noise are often located in close proximity to fully Polycomb-repressed genes, suggesting that heterochromatin spreading may play a role in producing noise, but the bursting parameters most affected by heterochromatin formation are still not understood.

Beyond Polycomb, little is known about the contributions of specific chromatin factors to transcriptional bursting. Recent work has examined the effect of chromatin environment on bursting by inserting transgenes into various locations within the genome of yeast and mammalian cells. Transcriptional burst size and frequency varies by insertion site, indicating that the local chromatin environment may shape bursting (Dar et al., 2012). However, the exact chromatin features that are responsible for these position effects are still unclear. Future studies could assess the effects of chromatin remodelers, pioneer transcription factors, histone modifications, and DNA marks in regulating transcriptional bursting and gene expression noise.

It is clear that enhancers and promoters play a role in regulating gene-specific bursting, while variation in DNA-polymerase interactions affect bursting genome-wide (Little et al., 2013; Fujita et al., 2016). Determining the molecular mechanisms of gene-specific and genome-wide bursting and elucidating how these two components are integrated *in vivo* is an area of intense study. With advances such as single molecule approaches, it is now possible to probe the effects of intrinsic gene expression noise on cellular heterogeneity and stochastic cell fate choices (Box 1). Next, we discuss the roles that transcriptional bursting and noise play in reproducible and stochastic specification of cell fates.

## How Noise Influences Cell Fate Specification

How does gene expression noise affect cell fate decisions during development? To ensure reproducible cell fates, developmental pathways must overcome noisy gene expression. Cells in reproducible lineages must therefore use noise buffering mechanisms to allow for proper pattern formation and reduce the effects of noise (Little et al., 2013). In contrast, isogenic populations of cells such as clonal populations of bacteria, harness gene expression noise as a mechanism to diversify cell fates (Balazsi et al., 2011). Stochastic cell fate decisions are also observed in multicellular organisms, but the mechanisms controlling these processes are still poorly understood.

### Noise Buffering in Deterministic Fate Choices

One of the most elegant examples of precise developmental patterning is the early *Drosophila* embryo. Expression patterns of the gap, segment polarity, and pair rule genes have been thoroughly mapped, but it was not until the development of smFISH and the MS2 hairpin system that the expression dynamics of these genes could be studied in greater detail (Garcia et al., 2013; Little et al., 2013; Gregor et al., 2014) (Box 1 and Figure 2).

**FIGURE 7:**
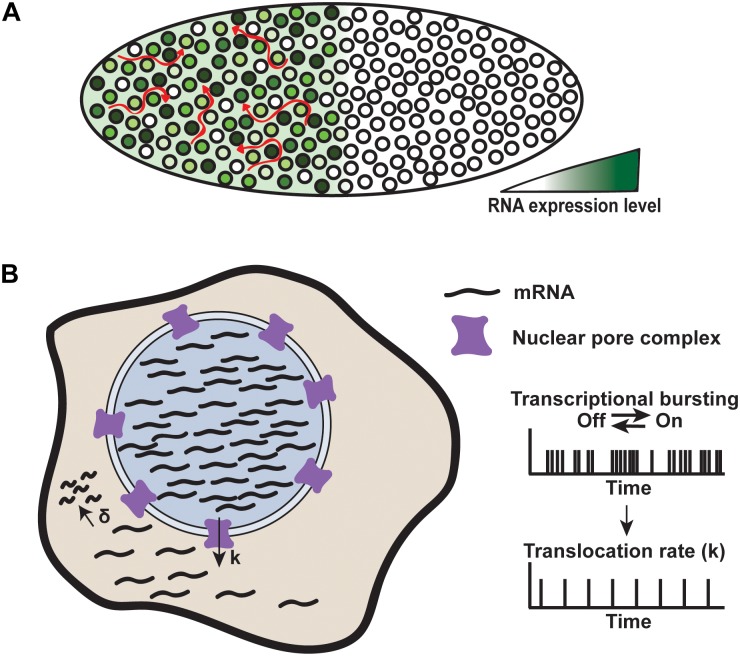
Mechanisms of noise buffering. **(A)** Transcriptional noise produced by the Hunchback gene is buffered in the *Drosophila* embryo. Each nucleus expresses Hunchback at varying levels, but RNA levels are buffered by spatiotemporal averaging across the cytoplasmic syncytium, denoted by red arrows. Adapted from Stoeger et al. (2016). **(B)** Nuclear retention of RNA acts to reduce cytoplasmic noise. RNA is produced through rapid bursts within the nucleus and translocated through the nuclear pour complex at a rate, k, which is slower than the RNA production rate. RNA is degraded at a rate, δ. Faster nuclear production compared to translocation constrains noise to the nucleus, while buffering cytoplasmic noise. Adapted from Battich et al. (2015).

With these techniques, it has been shown that the genes controlling early embryo patterning exhibit transcriptional bursting, producing gene expression noise (Gregor et al., 2007; Pare et al., 2009; Little et al., 2013; Bothma et al., 2014). The level of transcription at developmental patterning gene loci varies by about 45% between nuclei (Figure 7A). This level of noise is similar to that observed for non-developmental genes in *Drosophila*, which is surprising given that development is thought to be tightly regulated (Little et al., 2013). How is this high level of noise controlled to create a reproducible body plan?

During development, the early *Drosophila* embryo is a cytoplasmic syncytium, meaning that all of the mRNA produced by high- and low-expressing nuclei is transported into the same cytoplasmic environment. One mechanism by which noise is buffered in the early embryo is by mRNA diffusion throughout the syncytium, resulting in a spatiotemporal averaging of mRNA concentration (Figure 7A). This averaging dampens the noise produced by transcription from individual nuclei and allows for precise development (Little et al., 2013).

Although the cytoplasmic syncytium buffers a majority of the gene expression noise produced during early development, there are other forms of noise buffering that may act directly at the gene locus during transcriptional bursting. Many of the early *Drosophila* patterning genes are under the control of multiple enhancers, including back-up, or secondary enhancers, known as shadow enhancers, to ensure their precise spatial and temporal expression (Hong et al., 2008; Levine, 2010; Bothma et al., 2014, 2015). Indeed, a weak shadow enhancer can act additively with its primary enhancer to control transcriptional bursting and reduce expression noise (Bothma et al., 2015). Other forms of shadow enhancers, such as “dark” shadow enhancers promote robust expression patterns through the combinatorial use of enhancers and negative feedback (Yan et al., 2017).

Despite our knowledge of noise buffering in *Drosophila* embryos, understanding buffering in intact mammalian systems remains a challenge. Nevertheless, studies in cultured mouse cells and liver tissue using smFISH and single cell RNA-sequencing have begun to elucidate noise buffering in mammalian cells (Bahar Halpern et al., 2015; Battich et al., 2015; Stoeger et al., 2016). These studies have uncovered another potential mechanism by which cells buffer noise. For a subset of genes, noise is high within the nucleus but reduced in the cytoplasm, suggesting that, in addition to noise buffering by mRNA degradation, regulated mRNA export from the nucleus acts as a filter for noise (Figure 7B).

Thus, many layers of noise buffering exist to ensure reproducible cell fates despite large variability in gene expression between nuclei. Buffering occurs directly at the gene locus through the use of distinct functional enhancer and promoter elements, or post-transcriptionally through controlled nuclear export or averaging across a syncytium. Although not discussed in detail in this review, gene regulatory mechanisms such as negative feedback loops also reduce noise within larger regulatory systems (Raser and O’Shea, 2005; Chalancon et al., 2012).

### Noise Amplification Through Positive Feedback and Thresholding in Stochastic Cell Fate Decisions

In contrast to deterministic or reproducible cell fate specification where noise is buffered, stochastic cell fate specification utilizes transcriptional noise to generate diversity in cell fates. Some of the best-studied examples involve bet-hedging strategies. For example, within an isogenic population, a small subset of cells will take on a particular fate to diversify the population. These cells are said to be “hedging their bets” for the chance that the population can continue to survive and reproduce under stressful environmental conditions (Balazsi et al., 2011).

**FIGURE 8:**
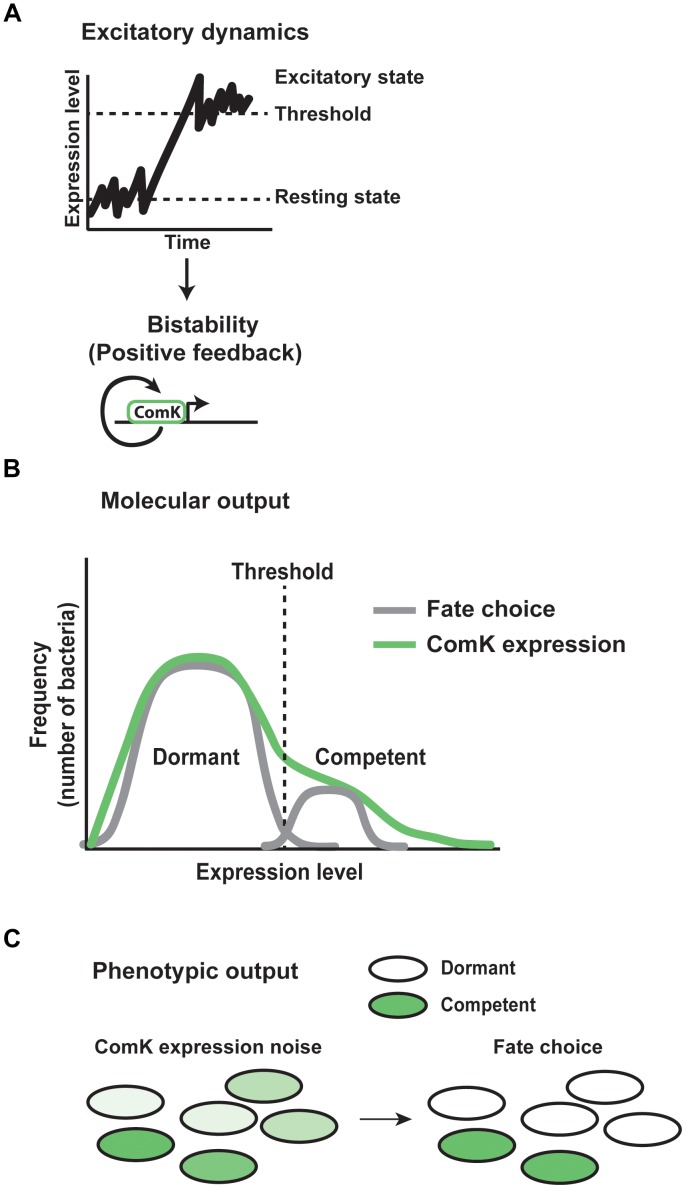
Mechanisms of stochastic cell fate specification. Gene expression noise plays a critical role in determining stochastic cell fates in the bacteria *Bacillus subtilis*. **(A)** In *Bacillus* populations, gene expression fluctuates between a resting and excitatory state. Excursions above a threshold level of expression induce bistability. A positive feedback loop for the protein ComK acts to stabilize the excitatory state of expression. **(B)** As a result of gene expression noise within the population of bacteria, the molecular output of excitatory dynamics and positive feedback are reflected by individual cells showing varying levels of ComK expression. Cells above threshold enter the competent fate, while cells below threshold remain in the dormant state. **(C)** Within the population, the phenotypic output of noise in ComK expression results in each individual expressing ComK at a different level. High expressing bacteria go on to assume the competent fate, while low expressing bacteria take on the dormant state.

The common mechanism underlying these decisions often involves bistable switching between high and low expression states. A high expression state occurs when gene expression spikes above a threshold in an individual cell. Cells that never reach threshold therefore remain in the resting state (Figure 8A). Once the threshold is surpassed, a positive feedback loop stabilizes high expression levels to maintain the cell fate (Figure 8A). In these systems, noise provides the gene expression variability necessary for switching, while positive feedback stabilize the fate decisions (Maamar et al., 2007; Balazsi et al., 2011).

One of the best-understood examples of stochastic cell fate choice involves the decision between competence and dormancy in *Bacillus subtilis*. Competence allows individuals to take in DNA from the environment that may provide a selective advantage to overcome stresses, at the risk of not surviving dangerous conditions. Noisy expression of the transcription factor ComK regulates this decision (Figure 8). Within a clonal population, each cell exhibits transcriptional bursting of ComK, producing noise between individual organisms (Mugler et al., 2016). In most cells, fluctuations in expression remain subthreshold (Figure 8B), resulting in a dormant cell fate. However, in a subset of cells, ComK expression surpasses a threshold, thereby inducing a switch to the competent fate (Figure 8C). At high levels of ComK, the protein binds directly to its own promoter as a dimer, stimulating its expression through a positive feedback loop (Figure 8A). This thresholding and feedback mechanism results in ∼10% of the bacterial population in the competent state at any given time (Maamar and Dubnau, 2005; Suel et al., 2006; Maamar et al., 2007).

A similar mechanism controls fate decisions for HIV-1. A key regulator of the HIV-1 life cycle is the transcription factor Trans-Activator of Transcription (Tat). If the expression of Tat reaches a certain threshold, its production will be stabilized through a positive feedback loop, thereby promoting active replication rather than proviral latency (Weinberger et al., 2005, 2008). Additionally, recent work on the mechanisms regulating Tat expression has shown that Tat is expressed in transcriptional bursts, supporting a role for an excitatory state being met through fluctuations in transcription, similar to that seen in bacteria (Weinberger et al., 2008; Hendy et al., 2017).

Stochastic fate choices also occur in multicellular organisms, but it is largely unclear how noise controls these decisions. Some preliminary work has been performed in hematopoietic cells, whose fates are influenced by the expression levels of the cell surface receptor Sca-1 (Chang et al., 2008). Noise in the levels of Sca-1 partitions these cells into high-expressing erythroid fates and low-expressing myeloid fates. However, the underlying mechanisms that stabilize these cell fates are not yet known. Future work may support the exciting possibility that the same general mechanisms guiding stochastic cell fates in bacteria and viruses are conserved in eukaryotic cells.

## Conclusion and Perspectives

Noise in gene expression can be utilized or buffered to control cell fates in both unicellular and multicellular organisms. Recent technological advances are allowing more in-depth studies of gene expression noise in multicellular organisms. Single molecule FISH and the MS2 hairpin system have been applied in several organisms, and the tracking of single RNA polymerases has been established *in vitro* (Lenstra et al., 2016). Future work developing single molecule live imaging to study noise and stochastic cell fates in other multicellular systems will provide important insights into the regulation of these processes in endogenous cellular contexts.

One potential system for future investigation is the *Drosophila* retina. Precise signaling cues produce a crystalline pattern of ommatidia (Wolff and Ready, 1991; Kumar, 2011). Despite this highly reproducible structure, patterning of R7 photoreceptor subtypes is stochastic. R7 cells randomly express the photopigment Rhodopsin 3 (Rh3) or Rhodopsin 4 (Rh4) (Bell et al., 2007; Johnston and Desplan, 2008, 2010). The decision to express Rh3 vs. Rh4 is controlled by the transcription factor Spineless (Ss), which is stochastically expressed in ∼70% of R7s, but the complete mechanism driving the on/off Ss decision is still unknown (Wernet et al., 2006; Johnston et al., 2011; Thanawala et al., 2013). Ss expression is partially controlled at the gene locus itself, which contains two repressing silencers and an activating enhancer (Johnston and Desplan, 2014). The transcriptional repressor Klumpfuss (Klu) also plays a critical role in determining the on/off ratio of Ss expression (Anderson et al., 2017). It is possible that the binary on/off output of Ss is achieved through noise in *ss* RNA levels during development in response to repression from Klu. For example, since transcription is inherently stochastic, and Klu binding to the *ss* locus will vary between cells, the level of repression in each cell will be noisy. If each cell expresses *ss* at a different level, this could imply that a particular threshold is necessary for producing the terminal decisions to be on or off by creating a bistable system. These observations raise the intriguing possibility that Ss is regulated similarly to ComK in *Bacillus subtilis* and Tat in HIV-1, suggesting a general paradigm for stochastic fate choice that is conserved through higher organisms.

Stochastic fate decisions are essential for development, pattern formation, and survival. The biological roles for noise in stochastic fate specification has been well-studied in viruses and bacteria, but its functions in eukaryotes are still being elucidated. With advances in quantitative imaging techniques, work on the roles of transcriptional bursting and noise in multicellular organisms is now accessible. Disruptions of stochastic fate specification mechanisms have been linked to human diseases including vision disorders, autism, anosmia, immunodeficiencies and lymphoma (Nathans, 1999; Morrow et al., 2008; Johnston and Desplan, 2010). Elucidating the roles for gene expression noise in cell fate decisions is therefore crucial for understanding when and how these systems go awry.

## Author Contributions

EU wrote and edited the manuscript. RJ edited the manuscript.

## Conflict of Interest Statement

The authors declare that the research was conducted in the absence of any commercial or financial relationships that could be construed as a potential conflict of interest.

## References

[B1] Anderson C. Reiss I. Zhou C. Cho A. Siddiqi H. Mormann B. (2017). Natural variation in stochastic photoreceptor specification and color preference in *Drosophila*. *eLife* 6:e29593. 10.7554/eLife.29593 29251595 PMC5745083

[B2] Bahar Halpern K. Tanami S. Landen S. Chapal M. Szlak L. Hutzler A. (2015). Bursty gene expression in the intact mammalian liver. *Mol. Cell* 58 147–156. 10.1016/j.molcel.2015.01.027 25728770 PMC4500162

[B3] Balazsi G. van Oudenaarden A. Collins J. J. (2011). Cellular decision making and biological noise: from microbes to mammals. *Cell* 144 910–925. 10.1016/j.cell.2011.01.030 21414483 PMC3068611

[B4] Bartman C. R. Hsu S. C. Hsiung C. C. Raj A. Blobel G. A. (2016). Enhancer regulation of transcriptional bursting parameters revealed by forced chromatin looping. *Mol. Cell* 62 237–247. 10.1016/j.molcel.2016.03.007 27067601 PMC4842148

[B5] Battich N. Stoeger T. Pelkmans L. (2015). Control of transcript variability in single mammalian cells. *Cell* 163 1596–1610. 10.1016/j.cell.2015.11.018 26687353

[B6] Bell A. C. West A. G. Felsenfeld G. (2001). Insulators and boundaries: versatile regulatory elements in the eukaryotic genome. *Science* 291 447–450. 10.1126/science.291.5503.447 11228144

[B7] Bell M. L. Earl J. B. Britt S. G. (2007). Two types of *Drosophila* R7 photoreceptor cells are arranged randomly: a model for stochastic cell-fate determination. *J. Comp. Neurol.* 502 75–85. 10.1002/cne.21298 17335038

[B8] Bertrand E. Chartrand P. Schaefer M. Shenoy S. M. Singer R. H. Long R. M. (1998). Localization of ASH1 mRNA particles in living yeast. *Mol. Cell* 2 437–445. 10.1016/S1097-2765(00)80143-4 9809065

[B9] Blake W. J. Balazsi G. Kohanski M. A. Isaacs F. J. Murphy K. F. Kuang Y. (2006). Phenotypic consequences of promoter-mediated transcriptional noise. *Mol. Cell* 24 853–865. 10.1016/j.molcel.2006.11.003 17189188

[B10] Bothma J. P. Garcia H. G. Esposito E. Schlissel G. Gregor T. Levine M. (2014). Dynamic regulation of eve stripe 2 expression reveals transcriptional bursts in living *Drosophila* embryos. *Proc. Natl. Acad. Sci. U.S.A.* 111 10598–10603. 10.1073/pnas.1410022111 24994903 PMC4115566

[B11] Bothma J. P. Garcia H. G. Ng S. Perry M. W. Gregor T. Levine M. (2015). Enhancer additivity and non-additivity are determined by enhancer strength in the *Drosophila* embryo. *eLife* 4:e07956. 10.7554/eLife.07956 26267217 PMC4532966

[B12] Buecker C. Wysocka J. (2012). Enhancers as information integration hubs in development: lessons from genomics. *Trends Genet.* 28 276–284. 10.1016/j.tig.2012.02.008 22487374 PMC5064438

[B13] Bulger M. Groudine M. (2011). Functional and mechanistic diversity of distal transcription enhancers. *Cell* 144 327–339. 10.1016/j.cell.2011.01.024 21295696 PMC3742076

[B14] Bury-Mone S. Sclavi B. (2017). Stochasticity of gene expression as a motor of epigenetics in bacteria: from individual to collective behaviors. *Res. Microbiol.* 168 503–514. 10.1016/j.resmic.2017.03.009 28427910

[B15] Cai L. Friedman N. Xie X. S. (2006). Stochastic protein expression in individual cells at the single molecule level. *Nature* 440 358–362. 10.1038/nature04599 16541077

[B16] Cairns B. R. (2009). The logic of chromatin architecture and remodelling at promoters. *Nature* 461 193–198. 10.1038/nature08450 19741699

[B17] Chalancon G. Ravarani C. N. Balaji S. Martinez-Arias A. Aravind L. Jothi R. (2012). Interplay between gene expression noise and regulatory network architecture. *Trends Genet.* 28 221–232. 10.1016/j.tig.2012.01.006 22365642 PMC3340541

[B18] Chang H. H. Hemberg M. Barahona M. Ingber D. E. Huang S. (2008). Transcriptome-wide noise controls lineage choice in mammalian progenitor cells. *Nature* 453 544–547. 10.1038/nature06965 18497826 PMC5546414

[B19] Chang S. Johnston R. J. Jr. Frokjaer-Jensen C. Lockery S. Hobert O. (2004). MicroRNAs act sequentially and asymmetrically to control chemosensory laterality in the nematode. *Nature* 430 785–789. 10.1038/nature02752 15306811

[B20] Chong S. Chen C. Ge H. Xie X. S. (2014). Mechanism of transcriptional bursting in bacteria. *Cell* 158 314–326. 10.1016/j.cell.2014.05.038 25036631 PMC4105854

[B21] Cochella L. Tursun B. Hsieh Y. W. Galindo S. Johnston R. J. Chuang C. F. (2014). Two distinct types of neuronal asymmetries are controlled by the *Caenorhabditis elegans* zinc finger transcription factor die-1. *Genes Dev.* 28 34–43. 10.1101/gad.233643.113 24361693 PMC3894411

[B22] Dar R. D. Razooky B. S. Singh A. Trimeloni T. V. McCollum J. M. Cox C. D. (2012). Transcriptional burst frequency and burst size are equally modulated across the human genome. *Proc. Natl. Acad. Sci. U.S.A.* 109 17454–17459. 10.1073/pnas.1213530109 23064634 PMC3491463

[B23] Dixon J. R. Gorkin D. U. Ren B. (2016). Chromatin domains: the unit of chromosome organization. *Mol. Cell* 62 668–680. 10.1016/j.molcel.2016.05.018 27259200 PMC5371509

[B24] Dixon J. R. Selvaraj S. Yue F. Kim A. Li Y. Shen Y. (2012). Topological domains in mammalian genomes identified by analysis of chromatin interactions. *Nature* 485 376–380. 10.1038/nature11082 22495300 PMC3356448

[B25] Dorsett D. (1993). Distance-independent inactivation of an enhancer by the suppressor of Hairy-wing DNA-binding protein of Drosophila. *Genetics* 134 1135–1144. 8375652 10.1093/genetics/134.4.1135 PMC1205581

[B26] Dowen J. M. Fan Z. P. Hnisz D. Ren G. Abraham B. J. Zhang L. N. (2014). Control of cell identity genes occurs in insulated neighborhoods in mammalian chromosomes. *Cell* 159 374–387. 10.1016/j.cell.2014.09.030 25303531 PMC4197132

[B27] Elowitz M. B. Levine A. J. Siggia E. D. Swain P. S. (2002). Stochastic gene expression in a single cell. *Science* 297 1183–1186. 10.1126/science.1070919 12183631

[B28] Enver T. Pera M. Peterson C. Andrews P. W. (2009). Stem cell states, fates, and the rules of attraction. *Cell Stem Cell* 4 387–397. 10.1016/j.stem.2009.04.011 19427289

[B29] Fudenberg G. Imakaev M. Lu C. Goloborodko A. Abdennur N. Mirny L. A. (2016). Formation of chromosomal domains by loop extrusion. *Cell Rep.* 15 2038–2049. 10.1016/j.celrep.2016.04.085 27210764 PMC4889513

[B30] Fujita K. Iwaki M. Yanagida T. (2016). Transcriptional bursting is intrinsically caused by interplay between RNA polymerases on DNA. *Nat. Commun.* 7:13788. 10.1038/ncomms13788 27924870 PMC5151093

[B31] Fukaya T. Lim B. Levine M. (2016). Enhancer control of transcriptional bursting. *Cell* 166 358–368. 10.1016/j.cell.2016.05.025 27293191 PMC4970759

[B32] Garcia H. G. Tikhonov M. Lin A. Gregor T. (2013). Quantitative imaging of transcription in living *Drosophila* embryos links polymerase activity to patterning. *Curr. Biol.* 23 2140–2145. 10.1016/j.cub.2013.08.054 24139738 PMC3828032

[B33] Geyer P. K. Corces V. G. (1992). DNA position-specific repression of transcription by a Drosophila zinc finger protein. *Genes Dev.* 6 1865–1873. 10.1101/gad.6.10.1865 1327958

[B34] Golding I. Paulsson J. Zawilski S. M. Cox E. C. (2005). Real-time kinetics of gene activity in individual bacteria. *Cell* 123 1025–1036. 10.1016/j.cell.2005.09.031 16360033

[B35] Gregor T. Garcia H. G. Little S. C. (2014). The embryo as a laboratory: quantifying transcription in *Drosophila*. *Trends Genet.* 30 364–375. 10.1016/j.tig.2014.06.002 25005921 PMC4129518

[B36] Gregor T. Tank D. W. Wieschaus E. F. Bialek W. (2007). Probing the limits to positional information. *Cell* 130 153–164. 10.1016/j.cell.2007.05.025 17632062 PMC2253670

[B37] Handoko L. Xu H. Li G. Ngan C. Y. Chew E. Schnapp M. (2011). CTCF-mediated functional chromatin interactome in pluripotent cells. *Nat. Genet.* 43 630–638. 10.1038/ng.857 21685913 PMC3436933

[B38] Hardy C. D. Cozzarelli N. R. (2005). A genetic selection for supercoiling mutants of *Escherichia coli* reveals proteins implicated in chromosome structure. *Mol. Microbiol.* 57 1636–1652. 10.1111/j.1365-2958.2005.04799.x 16135230

[B39] Harper C. V. Finkenstadt B. Woodcock D. J. Friedrichsen S. Semprini S. Ashall L. (2011). Dynamic analysis of stochastic transcription cycles. *PLoS Biol.* 9:e1000607. 10.1371/journal.pbio.1000607 21532732 PMC3075210

[B40] Hendy O. Campbell J. Jr. Weissman J. D. Larson D. R. Singer D. S. (2017). Differential context-specific impact of individual core promoter elements on transcriptional dynamics. *Mol. Biol. Cell* 28 3360–3370. 10.1091/mbc.E17-06-0408 28931597 PMC5687036

[B41] Hnisz D. Shrinivas K. Young R. A. Chakraborty A. K. Sharp P. A. (2017). A phase separation model for transcriptional control. *Cell* 169 13–23. 10.1016/j.cell.2017.02.007 28340338 PMC5432200

[B42] Hobert O. Johnston R. J. Jr. Chang S. (2002). Left-right asymmetry in the nervous system: the *Caenorhabditis elegans* model. *Nat. Rev. Neurosci.* 3 629–640. 10.1038/nrn897 12154364

[B43] Hong J. W. Hendrix D. A. Levine M. S. (2008). Shadow enhancers as a source of evolutionary novelty. *Science* 321:1314. 10.1126/science.1160631 18772429 PMC4257485

[B44] Hornung G. Bar-Ziv R. Rosin D. Tokuriki N. Tawfik D. S. Oren M. (2012). Noise-mean relationship in mutated promoters. *Genome Res.* 22 2409–2417. 10.1101/gr.139378.112 22820945 PMC3514670

[B45] Ing-Simmons E. Seitan V. C. Faure A. J. Flicek P. Carroll T. Dekker J. (2015). Spatial enhancer clustering and regulation of enhancer-proximal genes by cohesin. *Genome Res.* 25 504–513. 10.1101/gr.184986.114 25677180 PMC4381522

[B46] Jackson D. A. Pombo A. (1998). Replicon clusters are stable units of chromosome structure: evidence that nuclear organization contributes to the efficient activation and propagation of S phase in human cells. *J. Cell Biol.* 140 1285–1295. 10.1083/jcb.140.6.1285 9508763 PMC2132671

[B47] Johnston R. J. Jr. Chang S. Etchberger J. F. Ortiz C. O. Hobert O. (2005). MicroRNAs acting in a double-negative feedback loop to control a neuronal cell fate decision. *Proc. Natl. Acad. Sci. U.S.A.* 102 12449–12454. 10.1073/pnas.0505530102 16099833 PMC1194938

[B48] Johnston R. J. Jr. Copeland J. W. Fasnacht M. Etchberger J. F. Liu J. Honig B. (2006). An unusual Zn-finger/FH2 domain protein controls a left/right asymmetric neuronal fate decision in *C. elegans*. *Development* 133 3317–3328. 10.1242/dev.02494 16887832

[B49] Johnston R. J. Jr. Desplan C. (2008). Stochastic neuronal cell fate choices. *Curr. Opin. Neurobiol.* 18 20–27. 10.1016/j.conb.2008.04.004 18511260 PMC2478740

[B50] Johnston R. J. Jr. Desplan C. (2010). Stochastic mechanisms of cell fate specification that yield random or robust outcomes. *Annu. Rev. Cell Dev. Biol.* 26 689–719. 10.1146/annurev-cellbio-100109-104113 20590453 PMC3025287

[B51] Johnston R. J. Jr. Desplan C. (2014). Interchromosomal communication coordinates intrinsically stochastic expression between alleles. *Science* 343 661–665. 10.1126/science.1243039 24503853 PMC4134473

[B52] Johnston R. J. Jr. Hobert O. (2005). A novel *C. elegans* zinc finger transcription factor, lsy-2, required for the cell type-specific expression of the lsy-6 microRNA. *Development* 132 5451–5460. 10.1242/dev.02163 16291785

[B53] Johnston R. J. Jr. Otake Y. Sood P. Vogt N. Behnia R. Vasiliauskas D. (2011). Interlocked feedforward loops control cell-type-specific Rhodopsin expression in the *Drosophila* eye. *Cell* 145 956–968. 10.1016/j.cell.2011.05.003 21663797 PMC3117217

[B54] Johnston R. J. Hobert O. (2003). A microRNA controlling left/right neuronal asymmetry in *Caenorhabditis elegans*. *Nature* 426 845–849. 10.1038/nature02255 14685240

[B55] Kar G. Kim J. K. Kolodziejczyk A. A. Natarajan K. N. Triglia E. T. Mifsud B. (2017). Flipping between Polycomb repressed and active transcriptional states introduces noise in gene expression. *Nat. Commun.* 8:36. 10.1038/s41467-017-00052-2 28652613 PMC5484669

[B56] Kumar J. P. (2011). My what big eyes you have: how the *Drosophila* retina grows. *Dev. Neurobiol.* 71 1133–1152. 10.1002/dneu.20921 21604387 PMC3212655

[B57] Kumar J. P. (2012). Building an ommatidium one cell at a time. *Dev. Dyn.* 241 136–149. 10.1002/dvdy.23707 22174084 PMC3427658

[B58] Kumar N. Singh A. Kulkarni R. V. (2015). Transcriptional bursting in gene expression: analytical results for general stochastic models. *PLoS Comput. Biol.* 11:e1004292. 10.1371/journal.pcbi.1004292 26474290 PMC4608583

[B59] Larson D. R. Fritzsch C. Sun L. Meng X. Lawrence D. S. Singer R. H. (2013). Direct observation of frequency modulated transcription in single cells using light activation. *eLife* 2:e00750. 10.7554/eLife.00750 24069527 PMC3780543

[B60] Lenstra T. L. Rodriguez J. Chen H. Larson D. R. (2016). Transcription dynamics in living cells. *Annu. Rev. Biophys.* 45 25–47. 10.1146/annurev-biophys-062215-010838 27145880 PMC6300980

[B61] Lettice L. A. Heaney S. J. Purdie L. A. Li L. de Beer P. Oostra B. A. (2003). A long-range Shh enhancer regulates expression in the developing limb and fin and is associated with preaxial polydactyly. *Hum. Mol. Genet.* 12 1725–1735. 10.1093/hmg/ddg180 12837695

[B62] Levine M. (2010). Transcriptional enhancers in animal development and evolution. *Curr. Biol.* 20 R754–R763. 10.1016/j.cub.2010.06.070 20833320 PMC4280268

[B63] Lewis E. B. (1978). A gene complex controlling segmentation in *Drosophila*. *Nature* 276 565–570. 10.1038/276565a0 103000

[B64] Little S. C. Tikhonov M. Gregor T. (2013). Precise developmental gene expression arises from globally stochastic transcriptional activity. *Cell* 154 789–800. 10.1016/j.cell.2013.07.025 23953111 PMC3778922

[B65] Losick R. Desplan C. (2008). Stochasticity and cell fate. *Science* 320 65–68. 10.1126/science.1147888 18388284 PMC2605794

[B66] Ma H. Samarabandu J. Devdhar R. S. Acharya R. Cheng P. C. Meng C. (1998). Spatial and temporal dynamics of DNA replication sites in mammalian cells. *J. Cell Biol.* 143 1415–1425. 10.1083/jcb.143.6.1415 9852140 PMC2132991

[B67] Maamar H. Dubnau D. (2005). Bistability in the *Bacillus subtilis* K-state (competence) system requires a positive feedback loop. *Mol. Microbiol.* 56 615–624. 10.1111/j.1365-2958.2005.04592.x 15819619 PMC3831615

[B68] Maamar H. Raj A. Dubnau D. (2007). Noise in gene expression determines cell fate in *Bacillus subtilis*. *Science* 317 526–529. 10.1126/science.1140818 17569828 PMC3828679

[B69] Maduro M. F. (2010). Cell fate specification in the *C. elegans* embryo. *Dev. Dyn.* 239 1315–1329. 10.1002/dvdy.22233 20108317

[B70] McAdams H. H. Arkin A. (1997). Stochastic mechanisms in gene expression. *Proc. Natl. Acad. Sci. U.S.A.* 94 814–819. 10.1073/pnas.94.3.814 9023339 PMC19596

[B71] Morrow E. M. Yoo S. Y. Flavell S. W. Kim T. K. Lin Y. Hill R. S. (2008). Identifying autism loci and genes by tracing recent shared ancestry. *Science* 321 218–223. 10.1126/science.1157657 18621663 PMC2586171

[B72] Mugler A. Kittisopikul M. Hayden L. Liu J. Wiggins C. H. Suel G. M. (2016). Noise expands the response range of the *Bacillus subtilis* competence circuit. *PLoS Comput. Biol.* 12:e1004793. 10.1371/journal.pcbi.1004793 27003682 PMC4803322

[B73] Nasmyth K. (2011). Cohesin: a catenase with separate entry and exit gates? *Nat. Cell Biol.* 13 1170–1177. 10.1038/ncb2349 21968990

[B74] Nathans J. (1999). The evolution and physiology of human color vision: insights from molecular genetic studies of visual pigments. *Neuron* 24 299–312. 10.1016/S0896-6273(00)80845-4 10571225

[B75] Newlands S. Levitt L. K. Robinson C. S. Karpf A. B. Hodgson V. R. Wade R. P. (1998). Transcription occurs in pulses in muscle fibers. *Genes Dev.* 12 2748–2758. 10.1101/gad.12.17.2748 9732272 PMC317123

[B76] Nicolas D. Zoller B. Suter D. M. Naef F. (2018). Modulation of transcriptional burst frequency by histone acetylation. *Proc. Natl. Acad. Sci. U.S.A.* 115 7153–7158. 10.1073/pnas.1722330115 29915087 PMC6142243

[B77] Nora E. P. Lajoie B. R. Schulz E. G. Giorgetti L. Okamoto I. Servant N. (2012). Spatial partitioning of the regulatory landscape of the X-inactivation centre. *Nature* 485 381–385. 10.1038/nature11049 22495304 PMC3555144

[B78] Ozbudak E. M. Thattai M. Kurtser I. Grossman A. D. van Oudenaarden A. (2002). Regulation of noise in the expression of a single gene. *Nat. Genet.* 31 69–73. 10.1038/ng869 11967532

[B79] Pare A. Lemons D. Kosman D. Beaver W. Freund Y. McGinnis W. (2009). Visualization of individual Scr mRNAs during *Drosophila* embryogenesis yields evidence for transcriptional bursting. *Curr. Biol.* 19 2037–2042. 10.1016/j.cub.2009.10.028 19931455 PMC2805773

[B80] Peccoud J. Ycart B. (1995). Markovian modeling of gene-product synthesis. *Theor. Popul. Biol.* 48 222–234. 10.1006/tpbi.1995.1027

[B81] Poole R. J. Hobert O. (2006). Early embryonic programming of neuronal left/right asymmetry in *C. elegans*. *Curr. Biol.* 16 2279–2292. 10.1016/j.cub.2006.09.041 17141609

[B82] Postow L. Hardy C. D. Arsuaga J. Cozzarelli N. R. (2004). Topological domain structure of the *Escherichia coli* chromosome. *Genes Dev.* 18 1766–1779. 10.1101/gad.1207504 15256503 PMC478196

[B83] Racko D. Benedetti F. Dorier J. Stasiak A. (2018). Transcription-induced supercoiling as the driving force of chromatin loop extrusion during formation of TADs in interphase chromosomes. *Nucleic Acids Res.* 46 1648–1660. 10.1093/nar/gkx1123 29140466 PMC5829651

[B84] Raj A. Peskin C. S. Tranchina D. Vargas D. Y. Tyagi S. (2006). Stochastic mRNA synthesis in mammalian cells. *PLoS Biol.* 4:e309. 10.1371/journal.pbio.0040309 17048983 PMC1563489

[B85] Raj A. van Oudenaarden A. (2008). Nature, nurture, or chance: stochastic gene expression and its consequences. *Cell* 135 216–226. 10.1016/j.cell.2008.09.050 18957198 PMC3118044

[B86] Raser J. M. O’Shea E. K. (2004). Control of stochasticity in eukaryotic gene expression. *Science* 304 1811–1814. 10.1126/science.1098641 15166317 PMC1410811

[B87] Raser J. M. O’Shea E. K. (2005). Noise in gene expression: origins, consequences, and control. *Science* 309 2010–2013. 10.1126/science.1105891 16179466 PMC1360161

[B88] Ren G. Jin W. F. Cui K. R. Rodrigez J. Hu G. Q. Zhang Z. Y. (2017). CTCF-mediated enhancer-promoter interaction is a critical regulator of cell-to-cell variation of gene expression. *Mol. Cell* 67 1049–1058.e6. 10.1016/j.molcel.2017.08.026 28938092 PMC5828172

[B89] Ross I. L. Browne C. M. Hume D. A. (1994). Transcription of individual genes in eukaryotic cells occurs randomly and infrequently. *Immunol. Cell Biol.* 72 177–185. 10.1038/icb.1994.26 8200693

[B90] Sanborn A. L. Rao S. S. Huang S. C. Durand N. C. Huntley M. H. Jewett A. I. (2015). Chromatin extrusion explains key features of loop and domain formation in wild-type and engineered genomes. *Proc. Natl. Acad. Sci. U.S.A.* 112 E6456–E6465. 10.1073/pnas.1518552112 26499245 PMC4664323

[B91] Sarin S. O’Meara M. M. Flowers E. B. Antonio C. Poole R. J. Didiano D. (2007). Genetic screens for *Caenorhabditis elegans* mutants defective in left/right asymmetric neuronal fate specification. *Genetics* 176 2109–2130. 10.1534/genetics.107.075648 17717195 PMC1950618

[B92] Schuettengruber B. Bourbon H. M. Di Croce L. Cavalli G. (2017). Genome regulation by polycomb and trithorax: 70 years and counting. *Cell* 171 34–57. 10.1016/j.cell.2017.08.002 28938122

[B93] Seitan V. C. Faure A. J. Zhan Y. McCord R. P. Lajoie B. R. Ing-Simmons E. (2013). Cohesin-based chromatin interactions enable regulated gene expression within preexisting architectural compartments. *Genome Res.* 23 2066–2077. 10.1101/gr.161620.113 24002784 PMC3847776

[B94] Senecal A. Munsky B. Proux F. Ly N. Braye F. E. Zimmer C. (2014). Transcription factors modulate c-Fos transcriptional bursts. *Cell Rep.* 8 75–83. 10.1016/j.celrep.2014.05.053 24981864 PMC5555219

[B95] Sexton T. Yaffe E. Kenigsberg E. Bantignies F. Leblanc B. Hoichman M. (2012). Three-dimensional folding and functional organization principles of the *Drosophila* genome. *Cell* 148 458–472. 10.1016/j.cell.2012.01.010 22265598

[B96] Singh A. (2011). Negative feedback through mRNA provides the best control of gene-expression noise. *IEEE Trans. Nanobioscience* 10 194–200. 10.1109/TNB.2011.2168826 22020106

[B97] Skupsky R. Burnett J. C. Foley J. E. Schaffer D. V. Arkin A. P. (2010). HIV promoter integration site primarily modulates transcriptional burst size rather than frequency. *PLoS Comput. Biol.* 6:e1000952. 10.1371/journal.pcbi.1000952 20941390 PMC2947985

[B98] Sofueva S. Yaffe E. Chan W. C. Georgopoulou D. Vietri Rudan M. Mira-Bontenbal H. (2013). Cohesin-mediated interactions organize chromosomal domain architecture. *EMBO J.* 32 3119–3129. 10.1038/emboj.2013.237 24185899 PMC4489921

[B99] Spitz F. Furlong E. E. (2012). Transcription factors: from enhancer binding to developmental control. *Nat. Rev. Genet.* 13 613–626. 10.1038/nrg3207 22868264

[B100] Stigler J. Camdere G. O. Koshland D. E. Greene E. C. (2016). Single-molecule imaging reveals a collapsed conformational state for DNA-bound cohesin. *Cell Rep.* 15 988–998. 10.1016/j.celrep.2016.04.003 27117417 PMC4856582

[B101] Stoeger T. Battich N. Pelkmans L. (2016). Passive noise filtering by cellular compartmentalization. *Cell* 164 1151–1161. 10.1016/j.cell.2016.02.005 26967282

[B102] Suel G. M. Garcia-Ojalvo J. Liberman L. M. Elowitz M. B. (2006). An excitable gene regulatory circuit induces transient cellular differentiation. *Nature* 440 545–550. 10.1038/nature04588 16554821

[B103] Sulston J. E. Schierenberg E. White J. G. Thomson J. N. (1983). The embryonic cell lineage of the nematode *Caenorhabditis elegans*. *Dev. Biol.* 100 64–119. 10.1016/0012-1606(83)90201-4 6684600

[B104] Suter D. M. Molina N. Gatfield D. Schneider K. Schibler U. Naef F. (2011). Mammalian genes are transcribed with widely different bursting kinetics. *Science* 332 472–474. 10.1126/science.1198817 21415320

[B105] Swain P. S. (2004). Efficient attenuation of stochasticity in gene expression through post-transcriptional control. *J. Mol. Biol.* 344 965–976. 10.1016/j.jmb.2004.09.073 15544806

[B106] Swain P. S. Elowitz M. B. Siggia E. D. (2002). Intrinsic and extrinsic contributions to stochasticity in gene expression. *Proc. Natl. Acad. Sci. U.S.A.* 99 12795–12800. 10.1073/pnas.162041399 12237400 PMC130539

[B107] Takasuka N. White M. R. Wood C. D. Robertson W. R. Davis J. R. (1998). Dynamic changes in prolactin promoter activation in individual living lactotrophic cells. *Endocrinology* 139 1361–1368. 10.1210/endo.139.3.5826 9492073

[B108] Tantale K. Mueller F. Kozulic-Pirher A. Lesne A. Victor J. M. Robert M. C. (2016). A single-molecule view of transcription reveals convoys of RNA polymerases and multi-scale bursting. *Nat. Commun.* 7:12248. 10.1038/ncomms12248 27461529 PMC4974459

[B109] Thanawala S. U. Rister J. Goldberg G. W. Zuskov A. Olesnicky E. C. Flowers J. M. (2013). Regional modulation of a stochastically expressed factor determines photoreceptor subtypes in the *Drosophila* retina. *Dev. Cell* 25 93–105. 10.1016/j.devcel.2013.02.016 23597484 PMC3660048

[B110] Waddington C. H. (1957). The Strategy of the Genes: A Discussion of Some Aspects of Theoretical Biology. London: Allen and Unwin.

[B111] Weinberger L. S. Burnett J. C. Toettcher J. E. Arkin A. P. Schaffer D. V. (2005). Stochastic gene expression in a lentiviral positive-feedback loop: HIV-1 Tat fluctuations drive phenotypic diversity. *Cell* 122 169–182. 10.1016/j.cell.2005.06.006 16051143

[B112] Weinberger L. S. Dar R. D. Simpson M. L. (2008). Transient-mediated fate determination in a transcriptional circuit of HIV. *Nat. Genet.* 40 466–470. 10.1038/ng.116 18344999

[B113] Wernet M. F. Mazzoni E. O. Celik A. Duncan D. M. Duncan I. Desplan C. (2006). Stochastic spineless expression creates the retinal mosaic for colour vision. *Nature* 440 174–180. 10.1038/nature04615 16525464 PMC3826883

[B114] Wolf L. Silander O. K. van Nimwegen E. (2015). Expression noise facilitates the evolution of gene regulation. *eLife* 4:e05856. 10.7554/eLife.05856 26080931 PMC4468965

[B115] Wolff T. Ready D. F. (1991). The beginning of pattern formation in the Drosophila compound eye: the morphogenetic furrow and the second mitotic wave. *Development* 113 841–850. 1726564 10.1242/dev.113.3.841

[B116] Yan J. Anderson C. Viets K. Tran S. Goldberg G. Small S. (2017). Regulatory logic driving stable levels of defective proventriculus expression during terminal photoreceptor specification in flies. *Development* 144 844–855. 10.1242/dev.144030 28126841 PMC5374349

[B117] Zabidi M. A. Arnold C. D. Schernhuber K. Pagani M. Rath M. Frank O. (2015). Enhancer-core-promoter specificity separates developmental and housekeeping gene regulation. *Nature* 518 556–559. 10.1038/nature13994 25517091 PMC6795551

[B118] Zoller B. Little S. C. Gregor T. (2018). Diverse spatial expression patterns emerge from unified kinetics of transcriptional bursting. *Cell* 175 835–847.e5. 10.1016/j.cell.2018.09.056 30340044 PMC6779125

[B119] Zuin J. Dixon J. R. van der Reijden M. I. Ye Z. Kolovos P. Brouwer R. W. (2014). Cohesin and CTCF differentially affect chromatin architecture and gene expression in human cells. *Proc. Natl. Acad. Sci. U.S.A.* 111 996–1001. 10.1073/pnas.1317788111 24335803 PMC3903193

